# “Hiding in the Crowd”: defining conceptual models for privacy-preserving linkage of place-based and personal data for public benefit research.

**DOI:** 10.23889/ijpds.v9i5.2909

**Published:** 2024-09-18

**Authors:** Andy Boyd, Chris Dibben, Rich Fry, Pia Hardelid, Cassie Smith, Edel McNamara, Bethan Davies, Daniela Fecht, Ruth Blackburn, Ruth Gilbert

**Affiliations:** 1 University of Bristol; 2 University of Edinburgh; 3 Swansea University; 4 UCL; 5 Health Data Research UK; 6 Imperial College London

## Objective

To address the challenge of linking identifiable but low-sensitivity place-based data with high-sensitivity, de-identified, health and socio-economic records. While also minimising the financial and carbon costs of computationally intensive environmental modelling.

## Objective

For domain and legal experts, TRE leads and public contributors to co-develop a trusted system-wide governance model for linking these data.

## Results

We identified the UK’s ‘Unique Property Reference Numbers’ (UPRNs) - a geo-coordinated national property ID number - as ideally suited for linking individuals to households and households to place-based data. Under Data Protection laws, UPRNs are considered inherently identifiable, yet public-domain research use is permitted as the data have low sensitivity and there is broad societal acceptance. Once linked with individuals’ records, additional controls are needed to preserve privacy. We developed a workflow for modelling place-based data at a national level by a single agency and then, using trusted third parties, de-identifying and sharing minimised derived extracts into TREs for linkage and analysis. To maintain confidentiality, the UPRNs of the population of interest are masked by matched ‘control’ UPRNs drawn from the wider population. We will discuss permutations of the model, proof-of-concept implementations in UK TREs and public views on the models proposed.

## Conclusions

The challenge can be addressed by minimising the data flowing between national generators of place-based data and TREs using trusted third parties and masking data to maintain confidentiality.

## Implications

Relatively small adjustments to existing TRE workflows principles could enable new research possibilities without requiring re-evaluation of current governance norms or high-cost infrastructure changes.

